# Systematic Assessment of Clinical Methods to Diagnose and Monitor Diabetic Retinal Neuropathy

**DOI:** 10.1155/2018/8479850

**Published:** 2018-12-13

**Authors:** K. Sean Jenkins, Jason C. Steel, Christopher J. Layton

**Affiliations:** ^1^Faculty of Medicine, Greenslopes Clinical School, University of Queensland, Brisbane, Australia; ^2^LVF Ophthalmology Research Centre, Translational Research Institute, Brisbane, Australia; ^3^Central Queensland University, School of Health, Medical and Applied Science, Rockhampton, QLD, Australia

## Abstract

**Purpose:**

Diabetic retinal neuropathy refers to retinal neural tissue damage occurring before the structural retinal changes of diabetic retinopathy and fulfils many of the criteria for causality for the subsequent vasculopathy. Developing reliable means of measuring neuronal damage in diabetes may be important in efforts to prevent retinopathy of a clinically significant and irreversible stage. This study aimed at systematically assessing current clinical measurements of diabetic retinal neuropathy so that future studies may utilise a consensual battery of tests in studying this poorly understood disease state between a healthy retina and one that is retinopathic.

**Methods:**

A systematic search of the medical literature since 1984 was performed on PUBMED and EMBASE, and the evidence supporting each identified method as an indicator for clinically important diabetic retinal neuropathy was graded relatively as compelling, medium, or weak according to criteria assessing its relationship to subsequent diabetic retinopathy, quality of supporting studies, and published reproducibility.

**Results:**

The systematic search yielded 6432 results. Subsequent assessment by two independent investigators identified 601 multiple subject studies in humans assessing clinical aspects of the retinal structure, function, or psychophysics in the prediabetic retina. The 933 separate instances of clinical methods assessed as being supported by relatively “compelling” evidence included colour vision changes, flash ERG b-wave latency, flash multifocal b-wave latency, scotopic b-wave and oscillatory potentials in ERG, and contrast sensitivity.

**Conclusion:**

The results showed moderately poor quality of extant evidence and indicate the best clinical methods for assessing diabetic retinal neuropathy that remain to be confirmed. This is the first systematic assessment of the medical literature aiming at assessing the breadth and validity of these methods and represents an early step in identifying and developing clinical endpoints for use in trials designed to identify at-risk patients or prevent diabetic retinopathy.

## 1. Introduction

Diabetic retinopathy is a significant and progressive cause of blindness and visual impairment worldwide [1]. Diabetes causes panretinal damage over time via vasculopathic [2] and neuropathic [3] processes, eventually and progressively resulting in retinal ischaemia, haemorrhages, fibrosis, and irreversible blindness. Preceding clinically detectable retinopathy is a gradually progressive, not fully characterised, pre-retinopathic disease process described as diabetic retinal neuropathy [4–7]. Diabetic retinal neuropathy is referred to as a neurodegenerative precursor to diabetic retinopathy by multiple authors [4–6, 8]. To date, there has been little scientific consensus on the exact nature of this process, partly due to the inability to satisfactorily define it clinically or experimentally.

Due to its high metabolism, the retina is particularly vulnerable to the many metabolic changes that result from diabetes [5, 9]. Irreversible cumulative damage has been described in both the inner and outer retina [10], together with changes in glial cell morphology [9]; interruptions to the phototransduction cascade linked to early pericyte death [11, 12]; overproduction (or impaired removal) of lactate from a superfluity of glucose [5]; increased apoptosis and necrosis, possibly as a result of microglia-induced inflammation [6]; and retinal ischaemia [13]. Other changes that occur in the early diabetic retina that have been elucidated in animal models include but are not limited to thinning of the retinal outer nuclear layer (ONL) [7, 14–17]; apoptosis of ganglion cells [14–16]; hyperglycaemia and concomitant acidification of Muller cells [15, 18]; and changes to neuronal biochemistry, specifically hyperglycaemia; and dysregulation of neurotransmitter secretion [11, 12, 19]. In human psychophysical experiments, these changes to retinal anatomy and physiology in early diabetes are thought to contribute to electrophysiology changes [20–23], peripheral vision changes [4], colour vision loss [24], and loss of contrast sensitivity [24–26].

If the processes leading to this neurodegenerative precursor of clinical diabetic retinopathy could be clearly defined clinically using currently established or easily adopted testing procedures, it could facilitate further studies on potential treatments or preventative therapies against the development of diabetes-induced visual loss. Based on a systematic study of the body of the scientific literature using results from over 6400 papers to identify trends in the most useful clinical tests, this study provides an initial attempt to provide a meaningful clinical definition of diabetic retinal neuropathy according to easily performed clinical tests. Describing and quantifying a disease state that may be modifiable but not currently measurable in terms of the clinical tests that may be most useful in identifying the pattern of disease progression may allow further research into this disease process on a larger scale.

### 1.1. Approach

Investigation of retinal vasculopathy in diabetic retinopathy has remained the dominant scientific paradigm for many decades, and as such, the role and context of diabetic retinal neuropathy in the wider clinical problem of diabetes-induced blindness is less understood. Whilst the phenomenon is widely acknowledged, the field remains controversial: no one test has been shown to be the gold standard in diagnosing retinal neuropathy in diabetes. This study assumed that a system of assessments could better provide a scientifically based initial platform for identifying the condition and monitoring for both early disease progression and potential therapeutic effects in the interval before the traditional retinopathic stage of the disease.

This study therefore aimed first at systematically assessing the current best evidence behind commonly available clinical measurements of impaired neuronal structure or function in the retina in early diabetes. Secondarily, it aimed at analysing these results in the context of current clinical practice to inform the utility of commonly available functional indicators and tests of diabetic retinal neuropathy available to practicing ophthalmologists.

To maximize the characterisation of the current knowledge regarding clinically measurable early diabetic retinal changes, a critical analysis of known scientific evidence behind widely available clinical investigations of pre-retinopathic diabetic damage was conducted. However, further analysis to differentiate between the quality of evidence, clinical practicalities, and the risks and benefits of any proposed clinical test in the context of this systematic review of the literature was also considered in providing a framework to determine a justifiable appraisal of the available evidence for clinical assessment of the condition. Determining the baseline benefit of an investigation in the context of this immature field must be explicit and defensible. The internationally recognised GRADE system of evidence measurement satisfies all these criteria [27], and it is acknowledged as a reliable method of systematically and transparently assessing the benefits of a given set of investigations and for making recommendations based on those assessments [28, 29].

This article is the first to apply a system of assessing strength of evidence as a means of critically evaluating the large body of research on preclinical diabetic changes. This approach serves to define initial potential endpoints in diabetic retinal neuropathy for use within the context of other more established retinopathy grading systems, as there is currently no clear unequivocal measure of diabetic retinal neuropathy for use in a clinical or clinical trial setting.

This study uses a systematic review performed within a specific time frame. It uses the described search strategy and weighting system to sample the literature on this broad topic and proposes a framework of clinical tests to base further scholarship into this field upon. It is intended to be updated and refined in future, and because of its systematically collected nature, is necessarily is not exhaustive or comprehensive.

## 2. Materials and Methods

A systematic hand search of the medical literature since 1984 was performed by two blinded investigators on PUBMED and EMBASE. For a study to be considered, it required clinical testing of retinal function *in vivo* at a stage where patients were diabetic and did not demonstrate retinopathic changes. Studies which did not distinguish between patients with diabetes graded as nonretinopathic and early (mild or mod NPDR or equivalent) retinopathic grades were included. The evidence supporting each tested method likely to be clinically relevant for identifying diabetic retinal neuropathy was graded relatively as compelling, moderate, medium, or weak according to the GRADE criteria-based table (Table 1). The grade of evidence broadly assessed a positive clinical test's correlation with the development of subsequent diabetic retinopathy, quality of the study, and published reproducibility across studies; 6432 studies were identified, and 601 of these studies yielded 933 examples of metrics of assessing preclinical or clinical diabetic retinal changes. Search terms were designed in consultation with 3 evidence-based medicine experts and fell under three strings comprising clinical tests, retinal disease, and diabetes mellitus. Studies were excluded if they reported exclusively animal results, were published before 1984, were case reports, not published in English, used nonclinically or as functional means of assessing neuropathy including tissue samples, or were studies dealing with only extant clinical retinopathy. Interrater reliability of paper relevance and grading was 93.4%. Disagreements were resolved with consensus-based discussion of Furlan et al.'s study [33, 34].

**Figure 1:**
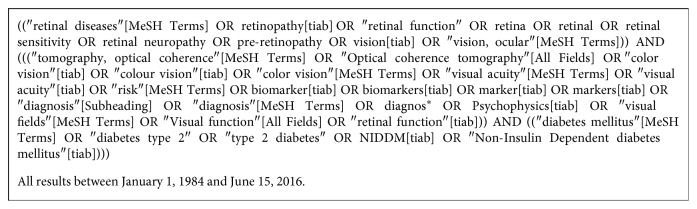
Database search string.

The search string used in PUBMED and EMBASE can be found in Figure 1.

Tabulation of strength of evidence criteria for an investigation is found in Table 1.

Note that a given investigation may have a compelling level of evidence in one study and a moderate level in another. This is due to differences in study design or strength of results, population size, and any statistical evidence of correlation. Similarly, multiple metrics may be present in a paper, with varying strengths depending on the focus and design of the study.

Assumptions have been made that together the clinical tests investigated in this study can increase the clinician's confidence due to functional changes in healthy diabetic subjects, structural diabetic changes are occurring to the retina in the absence of clinical signs, and these assumptions are to be tested in this and future studies. Potential risks of bias include selection bias through search term selection or study design, although feedback has been sought from multiple ophthalmic and academic experts to mitigate this where possible.

## 3. Results and Discussion

Analysis of tests that were involved in the prediction or monitoring of pre-retinopathic diabetic retinal changes was collated according to the number of studies it occurred in and the relative strength of the test as described above in the modified GRADE criteria and summarised in Supplementary Table 1 and broken into groupings of clinical tests in Tables 2 [Table tab3] [Table tab4]–5. As can be seen by the tables, a test can be shown to be compelling in one study and moderate or weak in others.

**Figure 2:**
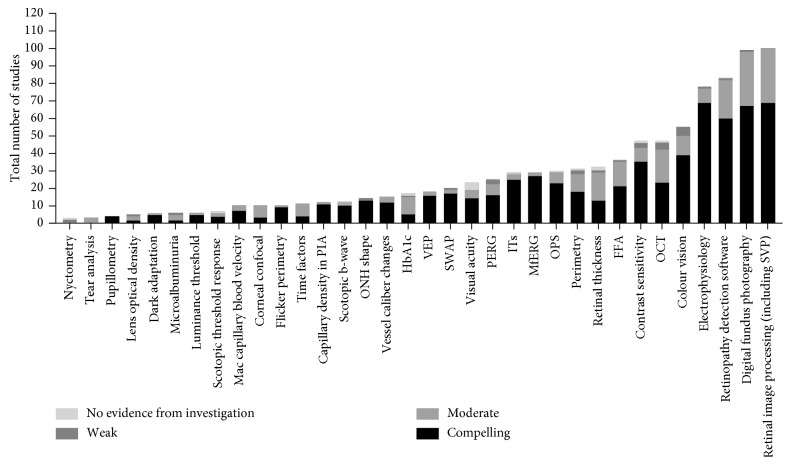
Total number of studies for each measure ranked according to strength of evidence. Retinal image processing, retinopathy detection software, and digital fundus photography have been employed most widely in the literature for detecting diabetic changes to the neural retina and are included where they have developed measures of diabetic retinal changes which are reported as preceding standard grades of diabetic retinopathy (such as vessel calibre changes, capillary density in perifoveal intercapillary space, and optic nerve head contour or shape). Unfortunately, they are flawed according to the GRADE criteria of this study in which there are no universal criteria or platform for these modalities in preclinical retinal neuropathy and lack criteria, nor do they indicate current nor future neural retina function.

Figure 2 shows all tests that were identified in the studies isolated in the systematic review for detecting pre-retinopathic diabetic changes. Almost all studies included some examples of clinical retinopathy, but most of them were focused on the first signs of clinical change and tested pre-retinopathic diabetic patients also. The final four rows will be interpreted separately from the preceding rows via subset analysis below. The ‘electrophysiology' row depicts the importance of all-source electrophysiology in detecting ophthalmoscopically invisible early derangements in retinal function, regardless of aetiology or the type of change. Retinopathy detection software and digital fundus photography are relevant for identifying the state of a diabetic retinopathy in the period at or after clinical detection of retinal retinopathy but are included where they have developed either proprietary or nonproprietary measures of early diabetic retinal changes which are reported as preceding standard grades of diabetic retinopathy, such as vessel calibre changes, capillary density in perifoveal intercapillary space, and optic nerve head contour or shape.

### 3.1. Electrophysiology

**Figure 3:**
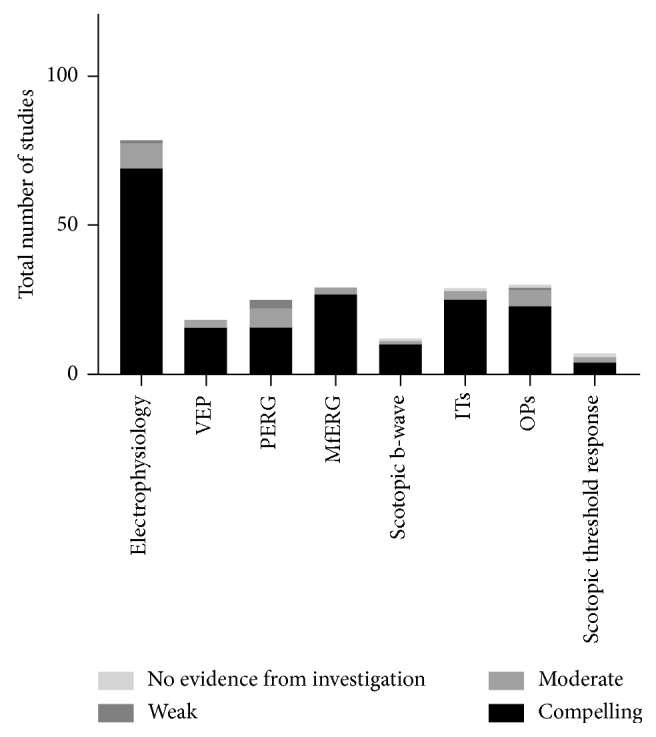
Number of studies of pre- and early diabetic retinopathy-detecting electrophysiology modalities, with each modality further sorted by number of studies at each relative strength of evidence. OPs have the greatest number of studies citing this test as evidence of retinal damage at 30, followed by ITs and mfERGs at 29 each. Proportionally, mfERG has the greatest amount of compelling evidence for retinal damage secondary to diabetes.

Generally, electrophysiology has a strong level of evidence in detecting pre-retinopathic diabetic neuropathy, especially via ERG oscillatory potentials (OPs) (Figure 3) [24, 35–37]. Multifocal ERG (mfERG) is more supported in the literature than visual-evoked potential (VEP) [13, 38, 39]. Examples of implicit times, oscillatory potentials, and scotopic b-waves being supported in studies as meaningfully measuring preclinical retinal neuropathy in human models can be found in Ewing et al. [24], Vadala et al. [36], Yoshida et al. [37], and Holopigian et al.[40], among many others.

### 3.2. Imaging

**Figure 4:**
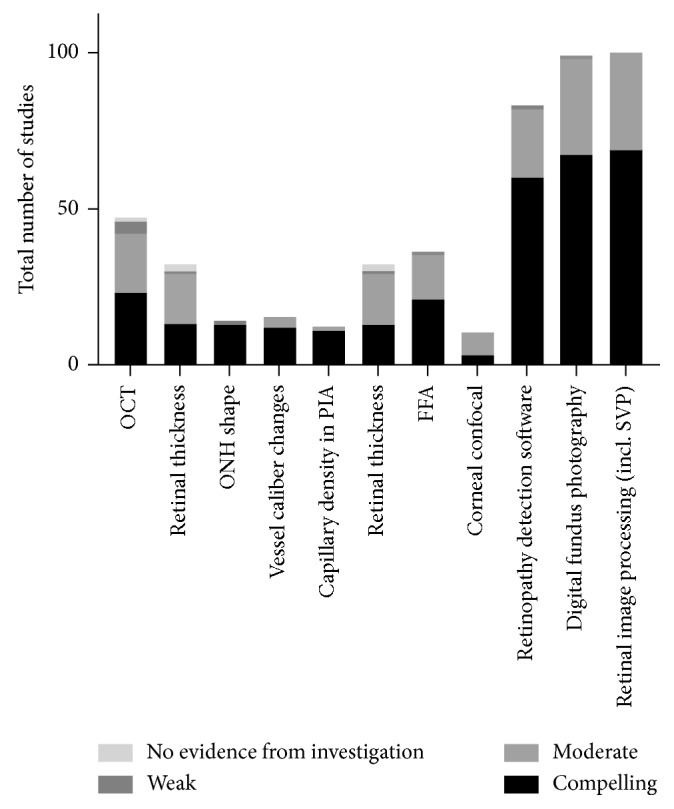
Number of studies of pre- and early diabetic retinopathy-detecting imaging modalities sorted by relative strength of evidence. OCT rather surprisingly does not have a strong base of compelling evidence for diabetic retinal neuropathy. The results for retinopathy detection software, digital fundus photography, and retinal image processing are ample, but problematic at this stage of inquiry into diabetic retinal neuropathy.

This study included methods of visualising diabetic changes in the retina. The limitations of these tests, though they are frequently cited in the literature, are that while they are a useful means of identifying end-stage damage to the neural retina vis-à-vis diabetic retinopathy, they give less useful insights into functional deficits of the neural retina at potentially reversible early stages, although relatively large numbers of results are reported at this stage. Additionally, significant disagreement over guidelines for imaging modalities and classification systems was found (Figure 4).

### 3.3. Functional Tests

**Figure 5:**
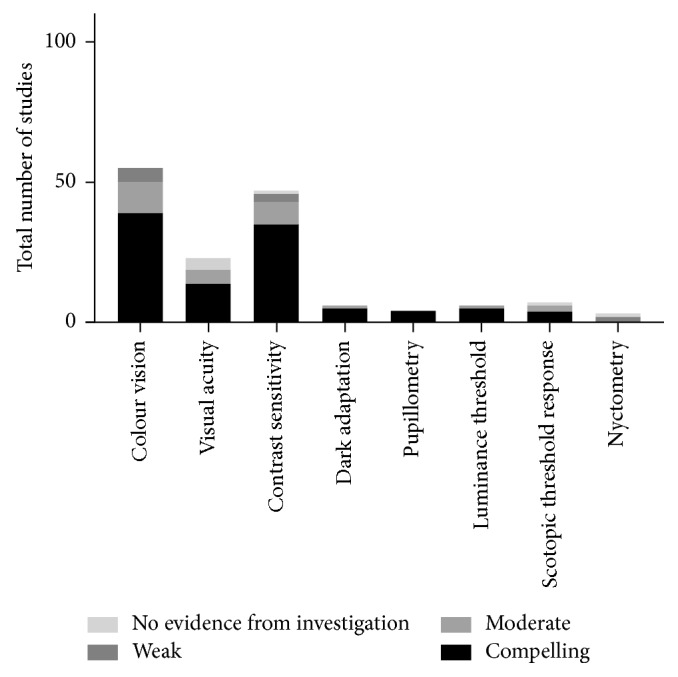
Number of studies of pre- and early diabetic retinopathy-detecting functional tests sorted by relative strength of evidence. These tests generally have greater variation in evidence strength than electrophysiology and imaging tests.

Contrast sensitivity and dark adaptation have more compelling evidence in the literature than clinical colour vision tests (the clear majority of which were performed with FM-100 colour tests) or best-corrected visual acuity (BCVA) for detecting early diabetic changes (Figure 5). As can be seen from the number of studies, pupillometry is a promising area for further study [41, 42] but currently lacks a sufficient body of evidence to make a recommendation on inclusion in retinal neuropathy investigation.

### 3.4. Perimetry

**Figure 6:**
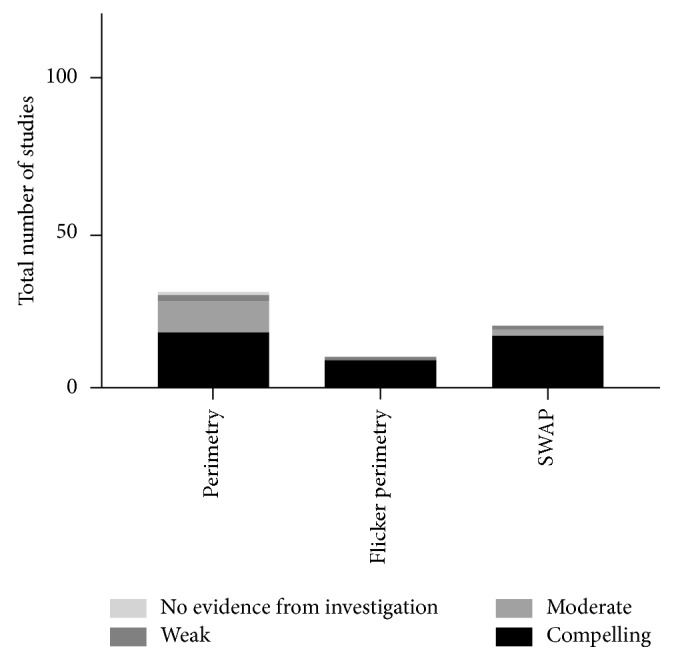
Number of studies of pre- and early diabetic retinopathy-detecting perimetric tests sorted by relative strength of evidence. Flicker perimetry and SWAP particularly make the argument for further usage in characterising and monitoring diabetic retinal neuropathy.

Perimetry has a well-established clinical role in monitoring subtle visual-field loss. Despite the relative paucity of studies, the significance of SWAP, a clinically archaic modality for glaucoma detection, proved to be interesting as a means of detecting early blue-yellow colour vision loss from reduced blue cone sensitivity in the diabetic retina [4], especially in young type 1 diabetic patients. Figure 2 and 6 demonstrate graphically the high proportion of compelling evidence for diabetic retinal changes in SWAP studies.

## 4. Discussion

After weighting the strength of evidence being examined, the second aspect of the GRADE system of assessment is the proposal of recommendations based upon the strength of evidence. For the purposes of this study, the clinical tests with the greatest proportion of compelling studies and the highest prevalence in the literature would be recommended in a battery of investigations to assess patients at risk of developing diabetic retinal neuropathy and, possibly, retinopathy.

Electrophysiology proved to have a relatively strong evidence base, with 69 cases of compelling evidence of detection of pre-retinopathic neuropathy. This included 27 cases of compelling evidence for mfERGs (therefore, out of 29, 93.1% of all mfERG cases were compelling), 25 for implicit times, both b-wave and 30 Hz flicker (86%), 23 for the magnitude of various combinations of oscillatory potentials (77%), and 16 for PERG and VEPs (64% and 89% of studies showed compelling evidence, respectively). There were only four cases compelling studies for scotopic threshold response, compared with two moderate and one case of no evidence (57%), but the small number of studies indicates further studies should be conducted rather than that this test does not have a value as a modality.

Ophthalmology is relatively unique amongst medical specialties in which pathology can be routinely visualised with the observer's own eyes in a routine clinical exam. Modalities include a simple retinal exam, OCT, and more advanced pattern recognition and processing software that mimics and seeks to improve upon a clinician's capacity for diabetic retinopathic pattern recognition. Great strides have been made in developing algorithms for analysing large numbers of diabetic retinas for frank retinopathy using the superior resolution capabilities of machines to detect diabetic changes such as microaneurysms or cotton-wool spots, but there is currently no consensus due to the proprietary nature of these technologies. Many studies showed compelling evidence for retinal imaging and computer processing software to detect early neuropathic changes as well, but as most were proprietary and not widely available, no analysis on widespread clinical utility could be made at this time, although the possibilities for development of this field are promising. Other imaging modalities, which showed good evidence of early diabetic changes, included vessel calibre changes (12 compelling, 3 moderate, 0 weak, or none), capillary density in perifoveal intercapillary space (11; 1; 0; 0), and optic nerve head shape (13; 0; 1; 0). OCT, obviously so useful in other fields including actual diabetic retinopathy, did not show a strong evidence base in this field, with retinal thickness measures (13; 16; 1; 2) being a relatively a poor metric for identifying or predicting retinal neuropathy. It is a possible new OCT, and adaptive optics technology could be more useful for this purpose in the future.

Several of the functional tests identified in this investigation are perhaps the most relevant to general clinical and clinical trial recommendations at the current stage of knowledge as they can be applied in any clinical setting, including general practice clinics and rural settings. The functional test with the most compelling evidence in the literature was contrast sensitivity (35; 8; 3; 1), which demonstrates early diabetic retinal changes, especially in young type 1 diabetics [25, 43]. Colour vision features strongly in the literature, although the evidence is more mixed, with 39 compelling studies, 11 moderate, 5 weak, but none with no evidence. Pupillometry (4; 0; 0; 0), dark adaptation (5; 1; 0; 0), luminance threshold (5; 1; 0; 0), scotopic threshold, (4; 2; 0; 1 as discussed above), and nyctometry (0; 1; 1; 1) all require further focused research due to the small number of studies identified; however, in individual studies, each appears to be promising as potential future indicators of retinal neuropathy investigations.

Perimetry also showed potential as a field of clinical investigation for detection of early diabetic changes and as a widely available test and a potentially clinically practical one. Perimetry (all types) had 18 cases of compelling evidence, 10 moderate, two weak, and only one study where evidence was not statistically significant. Short-wavelength automated perimetry (SWAP), despite its recently disappointing results in the glaucoma literature, has relatively strong scientific basis in detecting early diabetic changes (17; 2; 1; 0; 85% compelling). As young, type 1 diabetic patients theoretically have much longer to live with the disability of diabetic retinopathy, this modality's efficacy in early detection of retinal neuropathy merits revisiting it in a research and ultimately clinical setting as a possible valuable early therapeutic endpoint.

These findings are systematic, comprehensive, and allow the authors to make a series of recommendations for patients who are being assessed in clinical, clinical trial, and research settings for diabetic retinal neuropathy. As this is a developing field, during this investigation's inclusion period, several included studies were not specifically testing subjects for pre-retinopathic diabetic retinal neuropathy and patients with the early stages of diabetic retinopathy were included. The effect of this necessary methodological decision will likely become clearer once established protocols for quantifying diabetic retinal neuropathy in the absence of retinopathy are established, and more studies of retinal function are performed to specifically target the pre-retinopathic diabetic retina.

Patients at risk of diabetic retinopathy should receive the gold standard of diabetic retinopathy assessment according to local authority guidelines. In addition to a baseline BCVA being taken, patients should undergo contrast sensitivity and colour vision testing (the consensus in the literature reviewed here being that a FM-100 test or its derivatives, with computer grading assistance, is acceptable for clinical and research purposes [44]). Where possible, patients should also undergo electrophysiology, including some or all of mfERG, implicit times (30 Hz flicker and scotopic b-wave), and oscillatory potentials. Patients should undergo periodic flicker perimetry as well as SWAP, especially in young, type 1 diabetic patients. Though potential issues of cost exist, on the currently available evidence, this forms the scientifically strongest assessment of diabetic retinal neuropathy at this time.

It must be stressed that the study of diabetic retinal neuropathy is still an immature field. This initial attempt to categorically rate the strength of evidence of this disease process using a formal GRADE system is a systematic demonstration of current knowledge on the topic with a view to demonstrating the validity and usefulness of further study, as well as a providing a methodological framework for doing so. The data and conclusions found here will necessarily change with time and new data.

## 5. Conclusion

This exciting field of study is clearly in its infancy. Acknowledging the realities of clinical practice, modalities were selected that could make a real-world difference. Some of the decisions regarding the compelling nature of the literature were made selectively in the context of the entire literature review, comparing studies with one another. This comparative approach would not have been possible without an exhaustive and systematic review of the literature. This study reinforces many practices already employed by clinical ophthalmologists and codes a set of investigations that should be conducted at minimum in assessing diabetic patients for early neuropathic changes in the retina.

## Figures and Tables

**Table 1:** Strength of evidence (modified GRADE criteria).

Quality level	Definition
Compelling	Statistically significant relationship between a positive investigation result and the subsequent development of diabetic retinal pathology is shown in the study design. For a metric to be compelling, the study had to demonstrate a statistically significant predictability of change in the metric with the development of diabetic retinopathy

Indicative	Studies where the metric is used are either not shown to be statistically significant in the prediction of diabetic retinopathy but is still a useful clinical measure for presence and/or deterioration of retinopathy, or reviewers did not agree on the evidence of its compelling value

Moderate	Some evidence of a statistically significant relationship between a positive investigation result and the subsequent development of diabetic retinal pathology but problems with study design or applicability

Weak	Equivocal, unconvincing, and statistically insignificant correlation between investigation in the study and development of diabetic retinopathy

None	No evidence of correlation between investigation and development of diabetic retinopathy or contradictory evidence

A version of the GRADE criteria developed by Guyatt et al. [28–32] was created by the authors specific to ophthalmic interventions. Many ophthalmic clinical investigations are reliable and compelling due to their basis in psychophysics or their capacity for direct observation, and therefore, the terminology ‘compelling' is used rather than ‘strong', although definitive diabetic retinopathy cannot currently be demonstrated either via observation or through psychophysics with a single test.

**Table 2:** Number of studies with *electrophysiology* investigations demonstrating evidence of change in pre- or early diabetic retinopathy.

Electrophysiology	Compelling	Moderate	Weak	No evidence despite investigation
Electrophysiology total	69	8	1	0
VEP	16	2	0	0
PERG	16	6	3	0
MfERG	27	2	0	0
Scotopic b-wave	10	1	0	1
Implicit times (ITs)	25	3	0	1
Oscillatory potentials (OPs)	23	5	1	1

MfERG, OPs, and ITs had the greatest volume of evidence of diabetic retinal damage and, along with scotopic b-waves, the greatest proportion of compelling or indicative evidence of all electrophysiological tests.

**Table 3 tab3:** Number of studies with *imaging* investigations demonstrating evidence of changes before traditionally graded retinopathy.

Imaging	Compelling	Moderate	Weak	No evidence despite investigation
OCT	23	19	4	1
Retinal thickness	13	16	1	2
ONH shape	13	0	1	0
Vessel calibre changes	12	3	0	0
Capillary density in perifoveal intercapillary area	11	1	0	0
Retinal thickness	13	16	1	2
FFA	21	14	1	0
Corneal confocal	3	7	0	0
Retinopathy detection software	60	22	1	0
Digital fundus photography	67	31	1	0
Retinal image processing (including SVP)	69	31	0	0

While retinopathy detection software, digital fundus photography, and retinal image processing all had strong levels of indicative, if not compelling evidence for diabetic changes within the parameters of individual studies, there is little agreement on software or algorithm usage. Furthermore, in many cases, evidence was of early-stage clinical diabetic retinopathy, not preclinical retinal neuropathy. While OCT is an excellent imaging modality for many purposes in evaluating retinal health, relatively it was not a reliable indicator of diabetic retinal neuropathic changes in the literature.

**Table 4 tab4:** Number of studies with *functional* investigations demonstrating evidence of change in pre- or early diabetic retinopathy.

Functional tests	Compelling	Moderate	Weak	No evidence despite investigation
Colour vision	39	11	5	0
Visual acuity	14	5	0	4
Contrast sensitivity	35	8	3	1
Dark adaptation	5	1	0	0
Pupillometry	4	0	0	0
Luminance threshold	5	1	0	0
Scotopic threshold response	4	2	0	1
Nyctometry	0	1	1	1

The tests fulfil the functional assessment that imaging modalities lack and can lend some compelling weight to further retinal damage. However, there is far more variation in the strength of evidence for pre-retinopathic diabetic disease because the psychophysical margins of change at early stages are so small. Contrast sensitivity and colour vision tests have ample compelling and indicative evidence for inclusion into retinal neuropathic inquiry.

**Table 5:** Number of studies with *perimetry* investigations demonstrating evidence of change in pre- or early diabetic retinopathy.

Perimetry	Compelling	Moderate	Weak	No evidence despite investigation
Perimetry	18	10	2	1
Flicker perimetry	9	0	1	0
SWAP	17	2	1	0

Perimetry in general, and particularly 30 Hz flicker, and SWAP have generally very compelling or indicative evidence for demonstration of retinal neuropathic changes as a proportion of total studies addressing these modalities.

## Supplementary Materials

Supplementary Materials Supplementary Table 1: number of studies demonstrating evidence of change in pre- or early diabetic retinopathy (total raw data). The first numeric column demonstrates the total number of times a metric was used to demonstrate damage to the pre-retinopathy diabetic retina. The subsequent columns demonstrate the division of that number into instances of compelling, indicative, moderate, weak, and no significant evidence of neural retinal damage.
