# Supplementary material for: Systematic Assessment of Clinical Methods to Diagnose and Monitor Diabetic Retinal Neuropathy
Source: J Ophthalmol. 2018 Dec 13;2018:8479850. doi: 10.1155/2018/8479850 (PMC6311844; doi:10.1155/2018/8479850)
Supplement: Supplementary Materials — Supplementary Table 1: number of studies demonstrating evidence of change in pre- or early diabetic retinopathy (total raw data). The first numeric column demonstrates the total number of times a metric was used to demonstrate damage to the pre-retinopathy diabetic retina. The subsequent columns demonstrate the division of that number into instances of compelling, indicative, moderate, weak, and no significant evidence of neural retinal damage. [file 8479850.f1.pdf]

Supplementary Data

Investigation or Measure	Number	Compelling	Moderate	Weak	No evidence despite investigation
Nyctometry	3	0	1	1	1
Tear Analysis	3	0	3	0	0
Pupillometry	4	4	0	0	0
Lens Optical Density	5	2	2	1	0
Dark Adaptation	6	5	1	0	0
Microalbuminuria	6	2	3	1	0
Luminance threshold	6	5	1	0	0
Scotopic threshold response	7	4	2	0	1
Mac capillary blood velocity	10	7	3	0	0
Corneal Confocal	10	3	7	0	0
Flicker Perimetry (30Hz)	10	9	0	1	0
Time factors	11	4	7	0	0
Capillary density in perifoveal intercapillary area	12	11	1	0	0
Scotopic b-wave	12	10	1	0	1
ONH shape	14	13	0	1	0
Vessel Caliber changes	15	12	3	0	0
HbA1c	17	5	10	1	1
VEP	18	16	2	0	0
SWAP	20	17	2	1	0
Visual Acuity	23	14	5	0	4
PERG	25	16	6	3	0
ITs	29	25	3	0	1
MfERG	29	27	2	0	0
Ops (Oscillatory Potentials)	30	23	5	1	1
Perimetry	31	18	10	2	1
Retinal thickness	32	13	16	1	2
FFA	36	21	14	1	0
Contrast Sensitivity	47	35	8	3	1

OCT	47	23	19	4	1
Colour Vision	55	39	11	5	0
Electrophysiology	78	69	8	1	0
Retinopathy detection software	83	60	22	1	0
Digital fundus photography	99	67	31	1	0
Retinal image processing (including SVP)	100	69	31	0	0

Supplementary Table 1 – Number of studies demonstrating evidence of change in pre- or early diabetic retinopathy (total raw data). The first numeric column demonstrates the total number of times a metric was used to demonstrate damage to the pre-retinopathy diabetic retina. The subsequent columns demonstrate the division of that number into instances of compelling, indicative, moderate, weak, and no significant evidence of neural retinal damage.
